# Evaluating the biomechanical effects of implant diameter in case of facial trauma to an edentulous atrophic mandible: a 3D finite element analysis

**DOI:** 10.1186/s13005-017-0139-z

**Published:** 2017-05-02

**Authors:** Aysa Ayali, Kani Bilginaylar

**Affiliations:** Department of Oral and Maxillofacial Surgery, Near East University, Faculty of Dentistry, Near East Boulevard, Nicosia Cyprus, 99138 Mersin 10, Turkey

**Keywords:** Mandible, Fracture, Dental implant, Overdenture, Finite element analysis

## Abstract

**Background:**

Rehabilitation using an implant supported overdenture with two implants inserted in the interforaminal region is the easiest and currently accepted treatment modality to increase prosthetic stabilization and patient satisfaction in edentulous patients. The insertion of implants to the weakend mandibular bone decreases the strength of the bone and may lead to fractures either during or after implant placement. The aim of this three dimensional finite element analysis (3D FEA) study was to evaluate the biomechanical effects of implant diameter in case of facial trauma (2000 N) to an edentulous atrophic mandible with two implant supported overdenture.

**Methods:**

Three 3D FEA models were simulated; Model 1 (M1) is edentulous atrophic mandible, Model 2 (M2), 3.5x11.5 mm implants were inserted into lateral incisors area of same edentulous atrophic mandible, Model 3 (M3), 4.3x11.5 mm implants were inserted into lateral incisors area of same edentulous atrophic mandible.

**Results:**

In M1 and M2 highest stress levels were observed in condylar neck, whereas highest stress values in M3 were calculated in symphyseal area.

**Conclusions:**

To reduce the risk of bone fracture and to preserve biomechanical behavior of the atrophic mandible from frontal traumatic loads, implants should be inserted monocortically into spongious bone of lateral incisors area.

## Background

Although dental implant placement has become a usual treatment in recent years, the treatment of patients with atrophic mandible is still challenging. In the moderately or severely resorbed edentulous mandible, rehabilitation using an implant supported overdenture with two implants inserted in the interforaminal region is the easiest treatment modality to increase prosthetic stabilization and patient satisfaction [1, 2]. Such surgical procedures are anticipated, however, complications can be seen such as infection, improper placement, neurosensory injury, bleeding and mandible fracture which has a reported occurrence rate of 0.2%. The rate of incidence seems to be low, but it leads to overwhelming outcomes such as malunion, non-union, paresthesia, osteomyelitis and prolonged functional and nutritional disturbances [3]. On the other hand, the mandible is the most common broken bone by cause of facial injuries with the ratio of 23–97% [4]. The insertion of implants to the weakend mandibular bone decreases the strength of the bone and may lead to fractures either during or after implant placement [5]. Numerous case reports of fractured athrophic mandible secondary to implant insertion were reported in the literature [3, 6–8].

The principal areas of mandibular fracture are located in the condylar neck, the body or the angle and the symphysis of the mandible. The biomechanical behaviour of the mandible is important to know to understand the mechanism of fractures and to optimize treatment scenarios [9]. Clinically, the pattern of mandible fracture is related to various causes such as intensity and direction of the force, location of the impact point, position of the mandible at the time of injury, biomechanical properties of the mandible, overlying soft tissue, and the presence of teeth [10]. Although many studies have been reported that focused on these topics, declarations relating to the impacts of implant number, diameter, design, and length on the weakening of the atrophic edontulous mandible are rare and not depend on biomechanical evidence.

The Finite Elemet Analysis (FEA) has now become widely accepted and non-invasive tool that provides valuable results to estimate different parameters of the complex biomechanical behaviour of mandible [11–13].

The aim of this three dimensional finite element analysis (3D FEA) study was to evaluate the biomechanical effects of implant diameter in case of facial trauma to an edentulous atrophic mandible with two implant supported overdenture.

## Methods

**Fig. 1:**
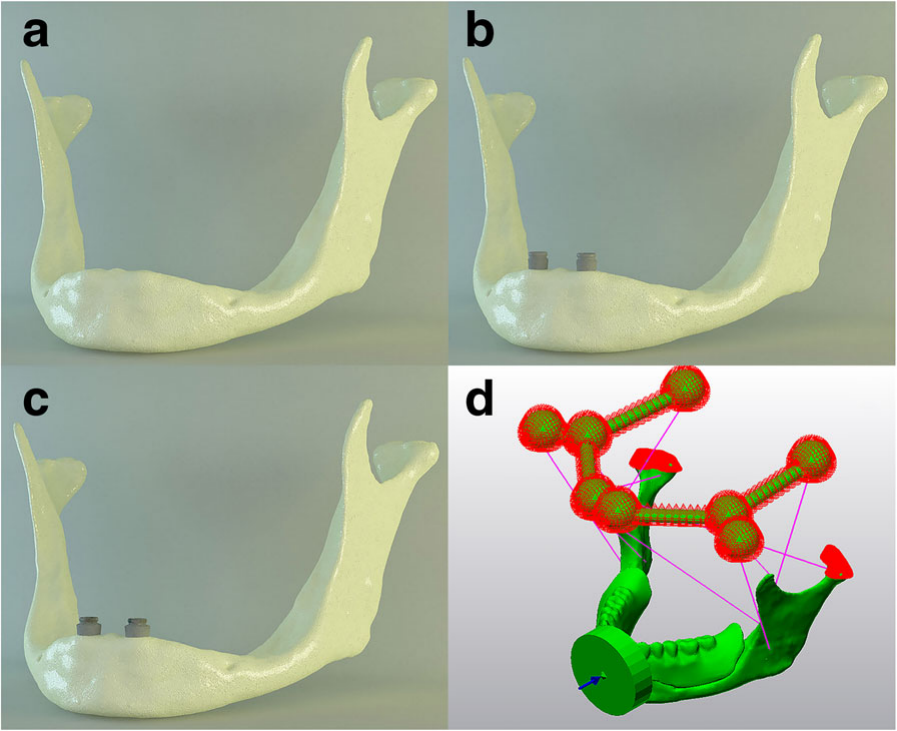
The loading and fixed boundary conditions of the models. **a**, M1. **b**, M2. **c**, M3. **d**

The three-dimensional models that were used in the current study were prepared with the help of single software to standardize all of the parameters of the models. Models were divided into three groups (Fig 1):

1. Model 1 (M1): Edentulous atrophic mandible (control model) (Fig 1a).
2. Model 2(M2): 3.5 × 11.5 mm Nobel Replace implants (Nobel Biocare USA, Yorba Linda, CA) were placed in the areas of both lateral incisors at a distance of 7 mm from the central point of the arch with the same vertical height level and Locator® attachments (Zest Anchors LLC, CA, USA) were used to connect implants to overdenture prosthesis (Fig 1b).
3. Model 3(M3): 4.3 × 11.5 mm Nobel Replace implants (Nobel Biocare USA, Yorba Linda, CA) were placed in the areas of both lateral incisors at a distance of 7 mm from the central point of the arch with the same vertical height level and Locator® attachments (Zest Anchors LLC, CA, USA) were used to connect implants to overdenture prosthesis (Fig 1c).

The data obtained from the Visible Human Project® (U.S. National Library of Medicine, Bethesda, MD, USA) were modified with the use of VRMESH (VirtualGrid Inc, Bellevue City, WA, USA) and Rhinoceros 4.0 (McNeel North America, Seattle, WA, USA) software to establish a 3D mandible FEA model to simulate clinical situation of edentulous atrophic mandible.

Mechanical properties of the materials that were simulated were taken from the literature [14–16] and are presented in Table 1. For standardization, the same overdentures were used by assuming that the material properties were the same for both the base part and artificial teeth. The implant-bone interface was considered to be static. The contact area of the overdenture and mucosa was assumed to be frictionless. ALGOR FEMPRO SOFTWARE (ALGOR Inc. Pittsburgh, PA, USA) was used to mesh final models with 3D parabolic tetrahedral solid elements with surface to surface contact. And then a refined mesh was performed in the mandible model to reproduce the compound stress formation observed in bone and implants. Total numbers of nodes and elements are listed in Table 2. Same software was also used to perform static analysis of the models.

**Table 1:** Mechanical properties of the materials

Material	Young’s Modulus (MPa)*	Poisson’s ration
Cortical Bone	13,700	0.3
Cancellous Bone	1,370	0.3
Titanium alloy	110,000	0.35
PMMA*	3,000	0.35
Mucosa	680	0.45

*Abbreviations: *MPa* Megapascal, *PMMA* Polymethyl methacrylate

**Table 2:** Total numbers of nodes and elements

Models	Nodes	Elements
Model 1	150440	691104
Model 2	282843	1339942
Model 3	280872	1319489

The mandibular condyles were fixed in all degrees of freedom. There are several muscles take place to close or elevate the mandible (Fig 1d). These muscles are masseter, temporal, medial and lateral pterygoid muscles. These muscles were modelled with no resistance during compression. Muscle tension stiffness values were reported previously in the literature: masseter muscle (16.35 N/mm), medial pterygoid muscle (15 N/mm), lateral pterygoid muscle (12 N/mm), anterior temporal muscle (14 N/mm) and posterior temporal muscle (13 N/mm) [10]. Traumatic force of 2000 N was applied perpendicularly to the frontal region on a 1 cm diameter circular area (Fig 1d). In previous FEA studies, force magnitude of 2000 N was used as a representative of a punch [10, 17].

After performing the FEA, maximum (Pmax) and minimum (Pmin) principle and Von Mises (VM) stresses were evaluated numerically and color coded.

## Results

### Von mises stresses in M1, M2 and M3 models

**Fig. 2:**
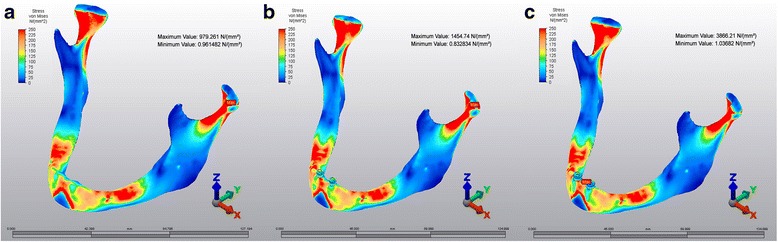
Von mises stress patterns. **a**: M1, **b**: M2, **c**: M3

The highest calculated values of Von Mises stresses in M1 (979.261 N/mm^2^) and M2 (1454.74 N/mm^2^) have been identified in the condylar area, whereas the highest value in M3 (3866 N/mm^2^) was observed in symphyseal area (Fig 2).

**Fig. 3:**
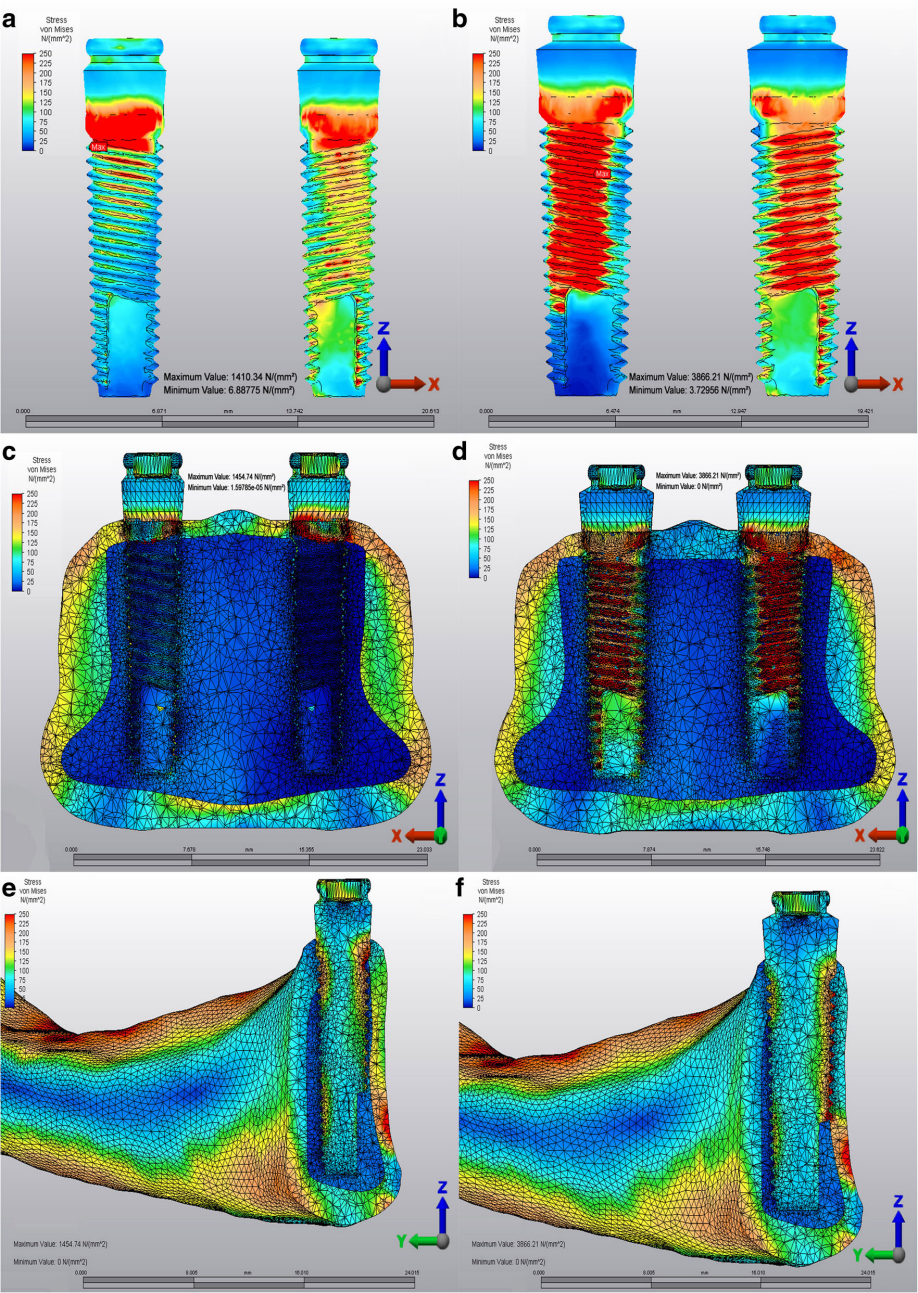
Von mises stress patterns of titanium implants. **a**: M2, **b**: M3, **c**: M2 frontal plane, **d**: M3 frontal plane, **e**: M2 sagittal plane, **f**: M3 sagittal plane

The evaluation of Von Mises stress patterns in the titanium implants of M3 showed that the stresses distributed all along the buccal surface of the implants, whereas in M2 high stresses were concentrated around the implant neck region (Fig 3).

### Pmax stresses in M1, M2 and M3 models

**Fig. 4:**
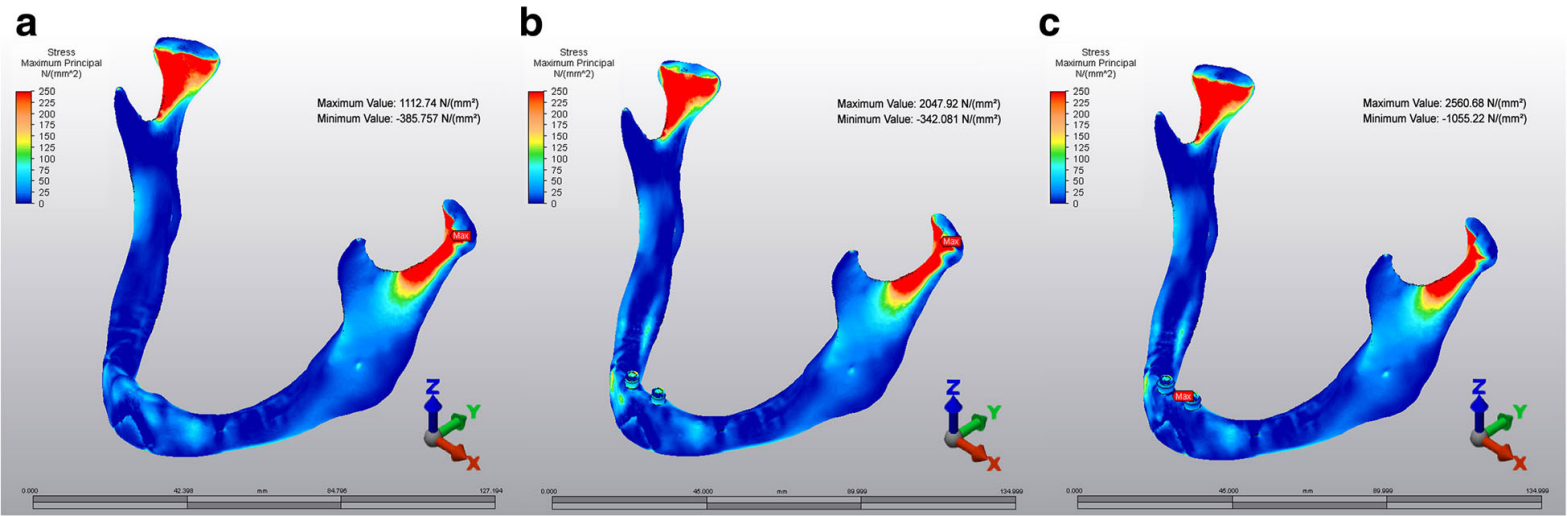
Pmax stress patterns. **a**: M1, **b**: M2, **c**: M3

The highest Pmax stress values in M1 (1112.74 N/mm^2^) and M2 (2047.92 N/mm^2^) were located in condylar area, whereas the highest value in M3 (2560.68 N/mm2) was observed in symphyseal area (Fig 4).

### Pmin stresses in M1, M2 and M3 models

**Fig. 5:**
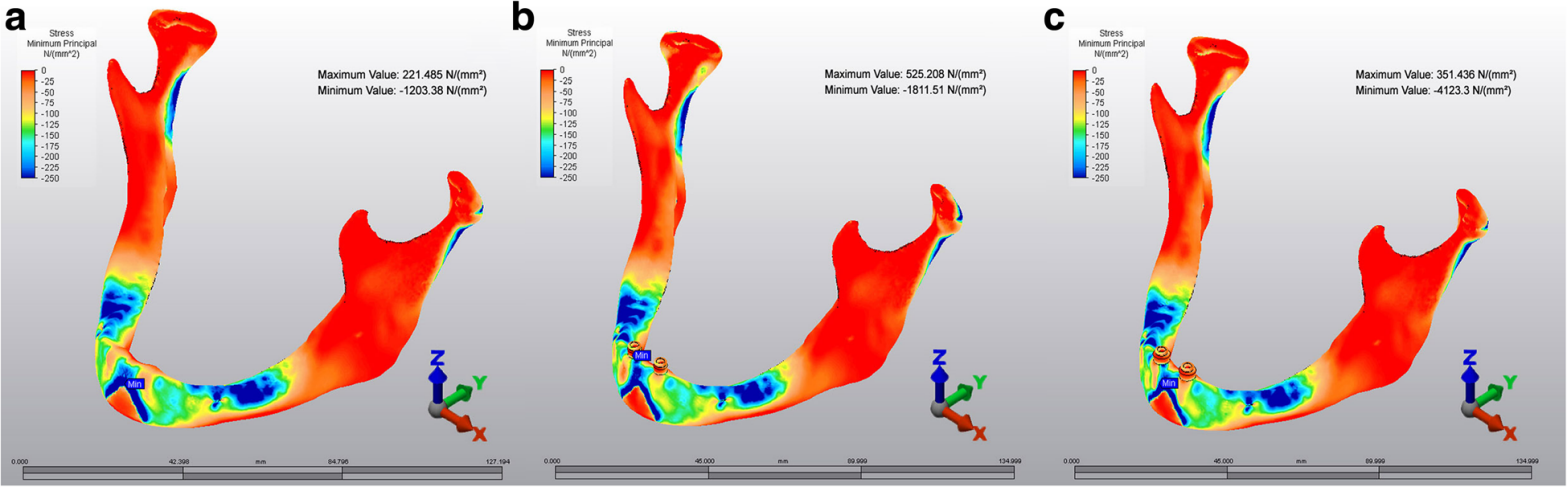
Pmin stress patterns. **a**: M1, **b**: M2, **c**: M3

The highest Pmin stress values were isolated in symphyseal area in all models.−1203.38 N/mm^2^,−1811.51 N/mm^2^ and−4125.3 N/mm^2^, respectively (Fig 5).

## Discussion

Patients that have atrophic edentulous mandibles suffer from psychosocial and functional problems related to their dentures. Insufficient stability and reduced retention of a lower denture because of a poor load−bearing capacity of the mandibular bone and remaining soft tissues hamper proper prosthodontic rehabilitation of these patients [7]. The insertion of two endosseous implants in the interforaminal region of an atrophic edentulous mandible is currently accepted and a widespread treatment option for improving retention and stability of a mandibular overdenture [5, 7, 18, 19].

Mandible fracture caused by endosseous implants was first reported by Albrektsson [20]. The ratio of a physician encountering a fractured atrophic mandible with dental implants has increased with the increase in use of dental implants [14]. Despite improvements in surgical fixation instruments, because of decreased vascularization, limited bone quality and quantity and absence of teeth in a patient with a fractured atrophic mandible, current procedures of fracture immobilization continues to be difficult and have been shown to be insufficient. Furthermore, fracture treatment is often complicated due to the poor health status and complex medical problems of older patients [21]. Clinicians involved in dental implant rehabilitation should realize that prevention is the best treatment for implant related mandibular fracture. That requires selection of patient, careful surgical technique, and postoperative care. Although such complications are rare, it is needed to be discussed preoperatively since the treatment of mandible fractures related to implants is complicated [3].

The aim of this tree-dimensional finite element analysis study was to evaluate the biomechanical behaviour of an atrophic mandible with two endoessous implants in response to traumatic force, based on differences in implant width. Additionally, this study will lead to find best insertion point of implants and selection of implant’s diameter, to prevent edentulous atrophic mandible fractures related to dental implants.

FEA is regarded as an adequate and convenient method for investigating stress and strain distribution by investigating the effect of the biomechanical properties of the bone and dental implants. It is difficult to assess the force distribution on jaws and dental implants due to the heterogeneous structure of the bones and the inability to simulate the effects of the muscles and soft tissues on the bones [14, 22, 23]. However, with the improvement of FEA software, dental implants and effects of soft tissues and muscles of the human jaws have demonstrated in the present study compatible with those of clinical situations. In the literature recent studies compared FEA analysis of mandible fracture with actual clinical cases and reported FEA to be an accurate, non-invasive, and repeatable method for studying the biomechanical behaviour of human mandibles under mechanical loads. Therefore, in ethical considerations FEA reduces the need for animal and cadaveric studies [9, 16, 22].

It was stated in the McGill (2002) and York (2009) Consensus Statements that a mandibular overdenture supported by two implants is the first choice of treatment for the mandibular edentulous patient [18, 19]. According to Kan et al. [14] placement of implants in the lateral incisor area is a better treatment modality than insertion in the canine area because of increase in inter-implant distance increases the fracture risk with in terms of frontal plane trauma. Previous clinical and biomechanical studies have reported that, long implant placement results in more stress to the implant and surrounding bone than short implant placement in the atrophic mandibles [5, 8, 14, 24]. In the present study 3.5 mm and 4.3 mm implant diameters have been used since these types of implants are widely regarded as standard diameter implants. Implants with narrow diametes of 3.0 to 3.25 mm are well documented only for single-tooth, non-load-bearing regions [25]. A meta-analysis study showed that narrower implants had significantly lower survival rates compared with wider implants [26]. Moreover, most authors advice at least 1 mm residual bone present to the adjacent to the implant surface [25]. Therefore, narrower and wider implant diameters were not used in the current study. For all these reasons, two monocortically inserted implants into lateral incisor areas of an atrophic mandible were simulated in the present study.

Previous FEA studies showed that the impact in the symphysis region of a dentate or edentulous mandible produced highest stress values in both the condylar neck areas which were similar with M1 model of current study [9, 16]. The highest calculated values of Von Mises stresses and Pmax stresses have been identified in the condylar area in M1 and M2, whereas the highest value in M3 was observed in symphyseal area. This could be because, there was spongious bone that may provide a homogenous stress distribution all around the implants in M2, whereas in M3 there wasn’t any spongious bone between implants and cortical bone on buccal side of implants. In M2, implants tend to tilt backwards in spongious bone and mandible moves to posteroinferior direction. Therefore, stresses accumulate at condylar neck areas. But in M3, because of absence of spongious bone on buccal side, implants are not able to move like in M2, therefore cortical bone transfers stresses directly to implants that makes the mandible to move only to posterior direction. That makes the stresses to distribute at the contact point of the implants to the bone (symphyseal area). Moreover in M2 high stress levels have been observed in implant neck area which is surrounded by only cortical bone. All these results showed that higher stress levels occur where implants directly come in contact with cortical bone. No experimental data can be found directly relevant to present study in the previous literatures to compare these results. Therefore, further studies in this area are needed.

## Conclusions

In conclusion, according to present study, to reduce the risk of bone fracture and to preserve biomechanical behavior of the atrophic mandible from frontal traumatic loads, implants should be inserted into lateral incisors area and into spongious bone monocortically.

## Abbreviations

3D: Three dimensional
FEA: Finite element analysis
Fig: Figure
M1: Model 1
M2: Model 2
M3: Model 3
MPa: Megapascal
N: Newton
N/mm: Newton/milimeter
N/mm^2^: Newton/milimeter square
Pmax: Maximum principle
Pmin: Minimum principle
PMMA: Polymethyl methacrylate
VM: Von mises
